# Development and Validation of a Nomogram to Predict the Future Risk of Cardiovascular Disease

**DOI:** 10.31083/j.rcm2402035

**Published:** 2023-01-31

**Authors:** Xuechun Shen, Wei He, Jinyu Sun, Zuhong Zhang, Qiushuang Li, Haiyan Zhang, Mingzhi Long

**Affiliations:** ^1^Department of Cardiology, The Second Affiliated Hospital of Nanjing Medical University, 210011 Nanjing, Jiangsu, China; ^2^Department of Geriatrics, The Second Affiliated Hospital of Nanjing Medical University, 210011 Nanjing, Jiangsu, China; ^3^Department of Cardiology, The First Affiliated Hospital of Nanjing Medical University, 210029 Nanjing, Jiangsu, China; ^4^Department of Technology, Nanjing Bottests Biotechnology Co, Ltd, 211112 Nanjing, Jiangsu, China

**Keywords:** biomarker, cardiac myosin-binding protein-C, cardiovascular disease, nomogram, risk prediction

## Abstract

**Background:**

Early identification of individuals at a high risk of cardiovascular disease (CVD) is crucial. This study aimed to construct a nomogram for CVD risk prediction in the general population.

**Methods:**

This retrospective study analyzed the data between January 2012 and September 2020 at the Physical Examination Center of the Second Affiliated Hospital of Nanjing Medical University (randomized 7:3 to the training and validation cohorts). The outcome was the occurrence of CVD events, which were defined as sudden cardiac death or any death related to myocardial infarction, acute exacerbation of heart failure, or stroke. The least absolute shrinkage and selection operator (LASSO) method and multivariate logistic regression were applied to screen the significant variables related to CVD.

**Results:**

Among the 537 patients, 54 had CVD (10.1%). The median cardiac myosin-binding protein-C (cMyBP-C) level in the CVD group was higher than in the no-CVD group (42.25 pg/mL VS 25.00 pg/mL, *p *= 0.001). After LASSO selection and multivariable analysis, cMyBP-C (Odds ratio [OR] = 1.004, 95% CI [CI, confidence interval]: 1.000–1.008, *p *= 0.035), age (OR = 1.023, 95% CI: 0.999–1.048,* p *= 0.062), diastolic blood pressure (OR = 1.025, 95% CI: 0.995–1.058, *p *= 0.103), cigarettes per day (OR = 1.066, 95% CI: 1.021–1.113, *p *= 0.003), and family history of CVD (OR = 2.219, 95% CI: 1.003–4.893, *p *= 0.047) were associated with future CVD events (*p <* 0.200). The model, including cMyBP-C, age, diastolic blood pressure, cigarettes per day, and family history of CVD, displayed a high predictive ability with an area under the curve (AUC) of 0.816 (95% CI: 0.714–0.918) in the training cohort (specificity and negative predictive value of 0.92 and 0.96) and 0.774 (95% CI: 0.703–0.845) in the validation cohort.

**Conclusions:**

A nomogram based on cMyBP-C, age, diastolic blood pressure, cigarettes per day, and family history of CVD was constructed. The model displayed a high predictive ability.

## 1. Introduction

Cardiovascular disease (CVD) is the leading cause of mortality globally [1]. China has a high burden of CVD, with a prevalence of 290 million, representing about 40% of the country’s mortality [2, 3]. Although treatments and secondary prevention improve the patient prognosis, primary prevention remains the best strategy but requires the early identification of individuals at a high risk of CVD. Over the last few decades, numerous CVD risk prediction models have been developed for the general population [4], including several well-known models, such as the Framingham model [5], SCORE [6], and QRISK3 [7]. Although many models exist for CVD risk prediction, their value remains unknown in China because of the differences in etiology and risk factors between Western and Chinese patients [8, 9]. More importantly, a notable characteristic of the CVD epidemiology in China is the rapidly increased mortality due to coronary heart disease (CHD) and the increasing age of the patients with CVD [10]. The analysis of Chinese data in the Global Burden of Disease study shows that fatal or nonfatal CHD events increased mainly in older adults aged 70–84 years [11]. Unfortunately, most CVD risk prediction models are not suitable for individuals aged >74 years. In addition, the association between traditional risk factors and CVD in older adults weakens with advancing age [12]. Therefore, an effective predictive biomarker beyond traditional risk factors needs to be explored to optimize the risk prediction model of CVD [13].

Recently, the studies by Saeed *et al*. [14] and Mehta *et al*. [15] reported that adding biomarkers (N-terminal pro-B-type natriuretic peptide, high-sensitivity cardiac troponin T, and high-sensitivity C-reactive protein) to the traditional risk factors improved CVD risk prediction in older adults. Increasing evidence shows that cardiac myosin-binding protein-C (cMyBP-C), a myocardium-restricted protein, can be used as an early diagnostic biomarker of acute myocardial infarction (MI) [16]. Large quantities of cMyBP-C are released into the bloodstream once a MI or ischemic injury occurs. Moreover, cMyBP-C is detectable in all healthy individuals independent of ischemic injury [17]. Incorporating plasma cMyBP-C levels in the analysis of CVD status and future events has been suggested as the most promising approach to achieve high prediction accuracy [18]. Still, whether cMyBP-C can be used as a predictive biomarker of CVD in Chinese patients is unknown.

Therefore, this study aimed to construct a nomogram for CVD risk prediction in the general population. This optimized model might demonstrate good performance for future CVD risk prediction in the general population, including young adults.

## 2. Materials and Methods

### 2.1 Study Design and Patients

This retrospective study analyzed the data between January 2012 and September 2020 at the Physical Examination Center of the Second Affiliated Hospital of Nanjing Medical University. This study was conducted following the Declaration of Helsinki (2013) and approved by the ethics committee of The Second Affiliated Hospital of Nanjing Medical University (No. [2021]-KY-078-01).

All individuals undergoing a medical examination between January 2012 and October 2013 were included. The exclusion criteria were (1) history of CVD (including MI, coronary insufficiency, angina, or heart failure), (2) active malignant tumor, or (3) missing data.

The patients were randomly assigned 7:3 to the training cohort for nomogram development and the validation cohort to confirm the model’s performance.

### 2.2 Data Collection and Outcome

The baseline characteristics and outcome of the patients were obtained from the electronic medical record system. Current smoking was defined as at least one cigarette a day for a stable period of 1 year [19]. Epidemiological information for cigarettes per day and family history of CVD was collected using a self-administered questionnaire. Body mass index [weight (kg)/height ${\mathrm{(m)}}^{2}$] was calculated. The definition of hypertension was systolic blood pressure (SBP) ≥140 mmHg or diastolic blood pressure (DBP) ≥90 mmHg or taking antihypertensive medication. Creatinine (Cr), uric acid (UA), triglycerides (TG), high-density lipoprotein cholesterol (HDL-C), and total cholesterol (TC) levels were measured routinely at the Department of Clinical Laboratory. Diabetes was defined as a fasting blood glucose ≥7.0 mmol/L (126 mg/dL), a diagnosis of diabetes, or the use of insulin or oral hypoglycemic medications. Blood samples were collected by standard ethylene diamine tetraacetic acid (EDTA) tubes and kept at room temperature for 30 min to allow clotting. After centrifugation at 3000 rpm for 15 minutes, the serum samples were frozen at –80 °C until cMyBP-C measurements were performed. All serum cMyBP-C levels were measured using sandwich enzyme-linked immunosorbent assay (ELISA) in a double-blinded manner. Specific cMyBP-C antibody (9B11C5) from GenScript was used to assess protein levels. The cMyBP-C levels were expressed as pg/mL. Neither storage at 4 °C for 3 days nor at –20 °C for 3 months resulted in any loss in immunoreactivity of the serum samples.

The outcome was the occurrence of CVD events, which were defined as sudden cardiac death or any death related to MI, acute exacerbation of heart failure, or stroke [20]. If patients declared the occurrence of CVD events, the medical records were verified. Final clinical event adjudication was based on the follow-up clinical contact or medical records, reached by a senior cardiologist blinded to clinical examination. The outcome classification was performed based on the International Classification of Diseases 10th revision (ICD-10). The inclusion criteria were I20 (angina), I21 (acute MI), and I24 (coronary insufficiency). The patients with a CVD event during follow-up were assigned to the CVD group and the others to the no-CVD group.

### 2.3 Statistical Analyses

The patients with a CVD event were assigned to the CVD group and the others to the no-CVD group. Categorical variables were presented as n (%), while continuous variables were presented as means ± standard deviation. The independent sample *t*-test was used to compare the continuous variables with a normal distribution (according to the Kolmogorov-Smirnov test), while the Wilcoxon rank-sum test was used to compare the continuous variables with a skewed distribution. The chi-square test was used to compare the categorical variables. Any variable having a significance level of *p <* 0.200 was retained for further feature selection [21].

The least absolute shrinkage and selection operator (LASSO) method and multivariate logistic regression was applied to screen the significant variables related to CVD (*p <* 0.200). Based on the above factors, a risk prediction model was constructed. At the same time, a nomogram model was built for quantitative estimation. In addition, a website was constructed to improve the availability of the nomogram.

The model’s predictive capability was assessed in the training cohort using the area under a receiver operating characteristic curve (AUC), specificity, and negative predictive value (NPV). The model accuracy was verified in the validation cohort. All statistical analyses were performed in R (version 3.6.1, R Foundation for Statistical Computing, Vienna, Austria).

## 3. Results

### 3.1 Characteristics of the Patients

**Fig. 1.**
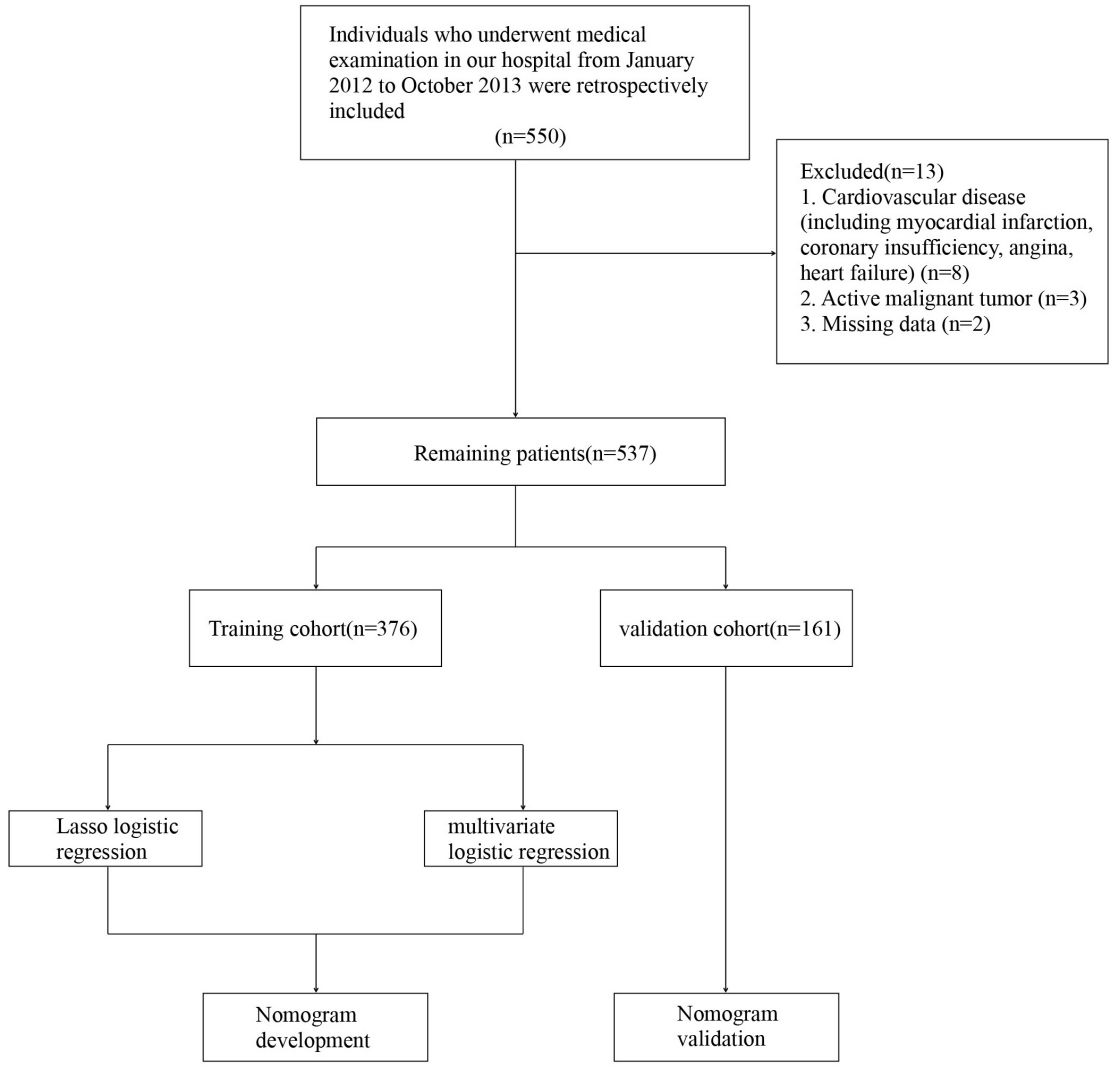
**Study flowchart**.

The study flowchart is shown in Fig. 1. Among the 537 patients, 54 had CVD (10.06%). As shown in Table 1, there were no significant differences in diabetes or UA between the CVD group (n = 54) and the no-CVD group (n = 483). The median cMyBP-C level was 42.25 pg/mL in the CVD group, which was significantly higher than in the no-CVD group (25.00 pg/mL). Pearson correlation analysis was performed to examine the association between age and cMyBP-C. As a result, the Pearson correlation coefficient was 0.01 (*p *= 0.73), indicating that cMyBP-C levels were not correlated with age. The patients in the training cohort were randomly assigned from 70% of all patients. The clinical characteristics of the training and validation cohorts were similar (**Supplementary Table 1**).

**Table 1.** **Baseline demographics and clinical characteristics**.

Variables	All patients (n = 537)	no-CVD group (n = 483)	CVD group (n = 54)	*p* value
cMyBP-C, pg/mL	26.50 [14.50, 47.50]	25.00 [14.00, 45.50]	42.25 [18.12, 67.75]	0.001
Age, years	49.00 [29.00, 61.00]	47.00 [28.00, 61.00]	59.00 [54.25, 64.75]	<0.001
Sex, female/male	301/236 (56.1/43.9)	276/207 (57.1/42.9)	25/29 (46.3/53.7)	0.168
BMI, kg/$m^{2}$	21.10 [19.40, 22.10]	21.10 [19.30, 22.00]	21.40 [19.75, 22.45]	0.122
SBP, mmHg	124.94 (16.01)	124.24 (15.90)	131.19 (15.75)	0.002
DBP, mmHg	76.00 [67.00, 82.00]	75.00 [67.00, 81.00]	80.00 [71.25, 85.75]	0.007
Hypertension, no/yes	468/69 (87.2/12.8)	429/54 (88.8/11.2)	39/15 (72.2/27.8)	0.001
Current smoking, no/yes	319/218 (59.4/40.6)	301/182 (62.3/37.7)	18/36 (33.3/66.7)	<0.001
Cigarettes per day	0.00 [0.00, 8.00]	0.00 [0.00, 6.00]	7.00 [0.00, 12.75]	<0.001
FBS, mmol/L	4.79 [4.46, 5.23]	4.78 [4.43, 5.23]	5.06 [4.55, 5.57]	0.012
Diabetes, no/yes	507/30 (94.4/5.6)	457/26 (94.6/5.4)	50/4 (92.6/7.4)	0.763
Cr, μmol/L	66.72 (15.00)	66.40 (14.90)	69.63 (15.79)	0.133
TC, mmol/L	4.42 [3.78, 4.99]	4.41 [3.76, 4.92]	4.61 [3.89, 5.29]	0.189
TG, mmol/L	1.69 [1.36, 1.88]	1.69 [1.36, 1.88]	1.77 [1.55, 1.88]	0.136
HDL-C, mmol/L	0.97 [0.86, 1.08]	0.97 [0.86, 1.10]	0.90 [0.80, 1.03]	0.011
UA, μmol/L	316.00 [257.00, 358.00]	317.00 [257.00, 357.50]	313.00 [262.75, 365.25]	0.977
Family history of CVD, no/yes	426/111 (79.3/20.7)	396/87 (82.0/18.0)	30/24 (55.6/44.4)	<0.001

CVD, cardiovascular disease; cMyBP-C, cardiac myosin-binding protein-C; BMI, body mass index; SBP, systolic blood pressure; DBP, diastolic blood pressure; FBS, fasting blood sugar; Cr, creatinine; TC, total cholesterol; TG, triglyceride; HDL-C, high-density lipoprotein cholesterol; UA, uric acid.

### 3.2 Construction of the Nomogram 

**Fig. 2.**
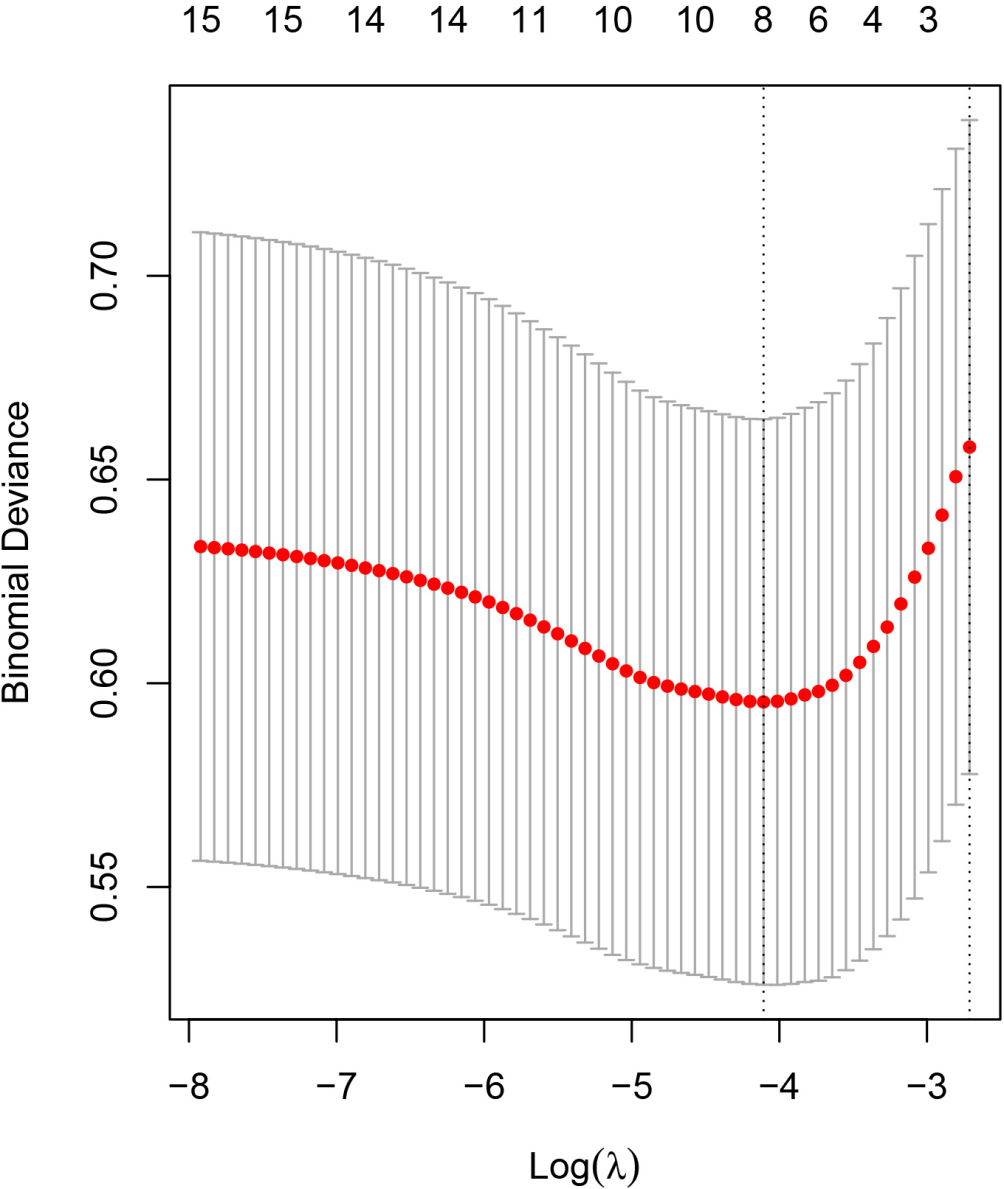
**Feature selection using the cross-validation least absolute shrinkage and selection operator (LASSO) binary logistic regression model**. Tuning parameter (λ) selection in the LASSO model used cross-validation via minimum criteria. A dotted vertical line was drawn at the value selected using cross-validation, where the optimal λ resulted in eight nonzero coefficients.

**Fig. 3.**
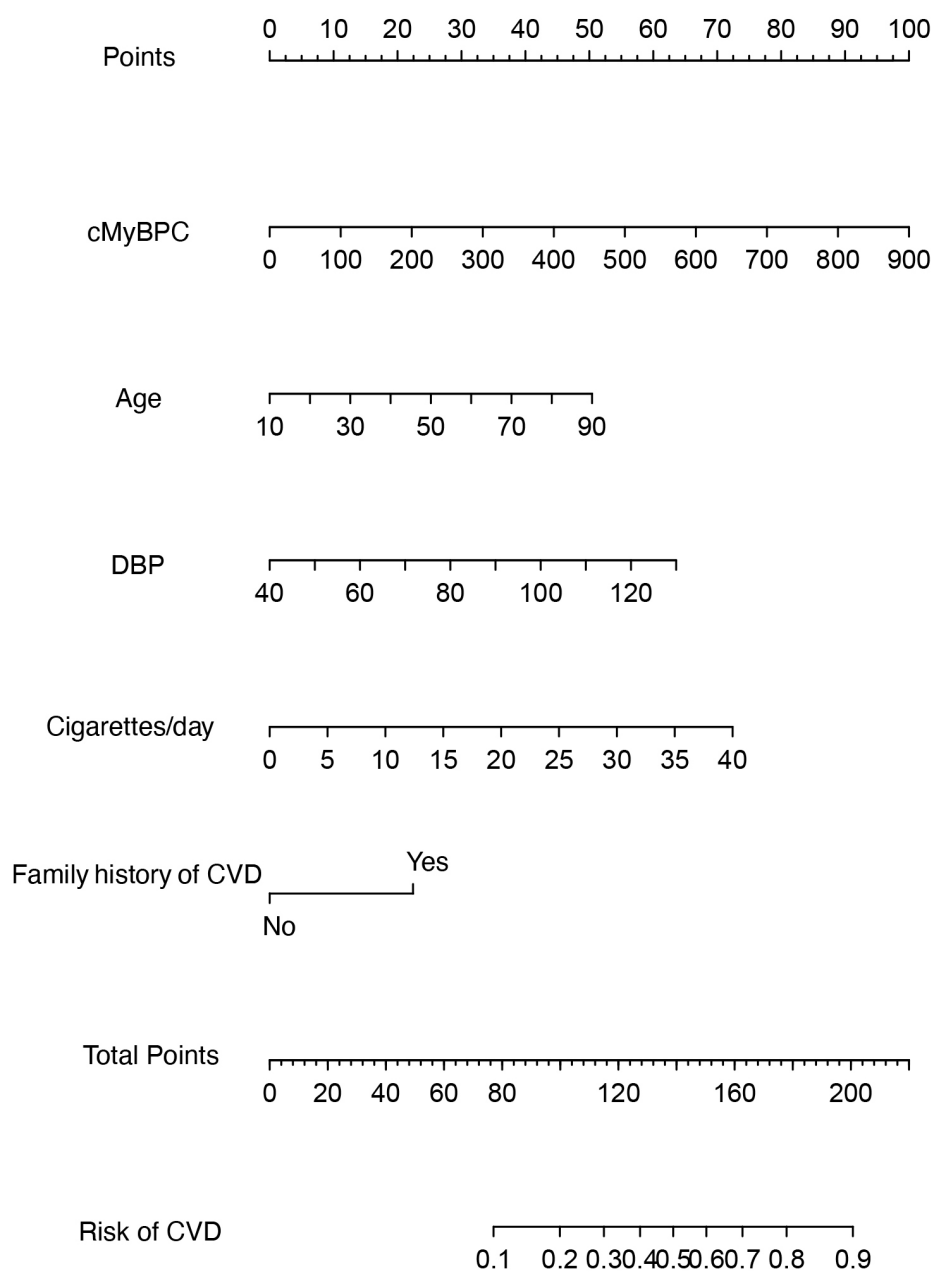
**Nomogram for the cardiovascular disease (CVD) risk prediction**. The nomogram was developed in the training cohort, incorporating cardiac myosin-binding protein-C (cMyBP-C), age, diastolic blood pressure (DBP), cigarettes per day, and family history of CVD.

In the training cohort, eight potential predictors feature with nonzero coefficients were selected from fifteen variables by the LASSO regression (Fig. 2): cMyBP-C, age, DBP, hypertension, current smoking, cigarettes per day, TG, and family history of CVD. HDL-C was retained artificially because of its clinical relevance in the epidemiology of CVD. All nine variables were entered into the multivariable logistic regression analysis. As shown in Table 2, only cMyBP-C, age, DBP, cigarettes per day, and family history of CVD were associated with future CVD events (*p *< 0.200). Next, the risk prediction model composed of the five independent predictors was developed (Table 3). In addition, a nomogram was constructed based on the model to provide a quantitative tool for estimating the risk of CVD (Fig. 3) .

**Table 2.** **Multivariable logistic regression analysis for feature selection**.

Variables	β	OR (95% CI)	*p* value
cMyBP-C	0.004	1.004 (1.000–1.008)	0.056
Age	0.022	1.022 (0.997–1.049)	0.090
DBP	0.025	1.025 (0.993–1.060)	0.129
Current smoking	0.194	1.214 (0.423–3.386)	0.713
Hypertension	0.022	1.022 (0.382–2.577)	0.964
Cigarettes per day	0.055	1.057 (0.993–1.122)	0.071
TG	0.172	1.188 (0.655–2.100)	0.561
HDL-C	–0.069	0.933 (0.138–5.222)	0.940
Family history of CVD	0.789	2.201 (0.992–4.873)	0.051

β, regression coefficient; OR, odds ratio; CI, confidence interval; cMyBP-C, cardiac myosin-binding protein-C; DBP, diastolic blood pressure; TG, triglyceride; HDL-C, high-density lipoprotein cholesterol; CVD, cardiovascular disease.

**Table 3.** **Coefficients and OR from the multivariable logistic regression model used for the CVD risk prediction model**.

Variables	β	OR (95% CI)	*p* value
cMyBP-C	0.004	1.004 (1.000∼1.008)	0.035
Age	0.022	1.023 (0.999∼1.048)	0.062
DBP	0.025	1.025 (0.995∼1.058)	0.106
Cigarettes per day	0.064	1.066 (1.021∼1.113)	0.003
Family history of CVD	0.797	2.219 (1.003∼4.893)	0.047

OR, odds ratio; CVD, cardiovascular disease; β, regression coefficient; CI, confidence interval; cMyBP-C, cardiac myosin-binding protein-C; DBP, diastolic blood pressure.

### 3.3 Performance and Validation of the Nomogram

**Fig. 4.**
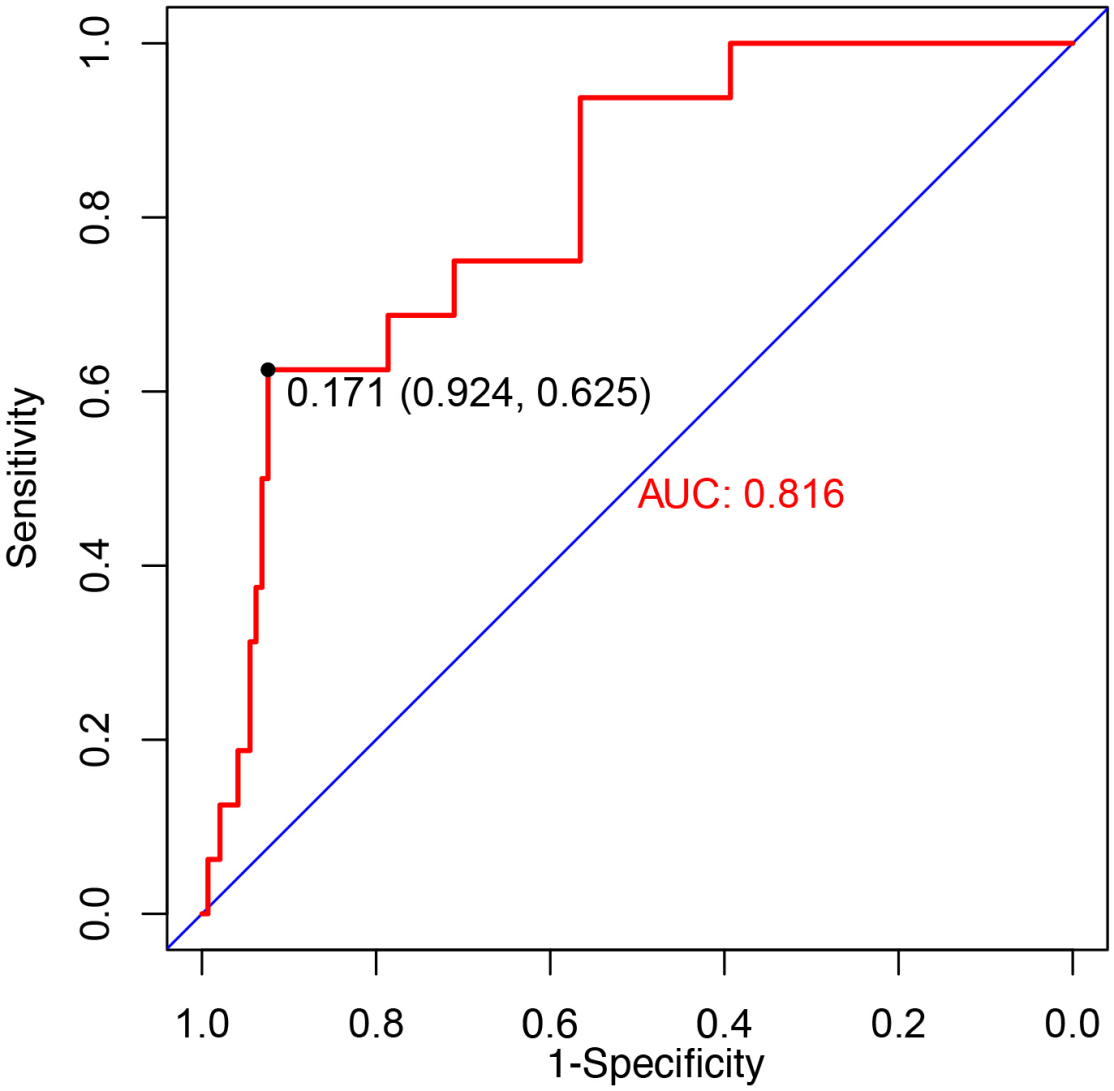
**Receiver operating characteristic (ROC) curve of the model**. The predictive performance of the cardiovascular disease (CVD) risk prediction model was evaluated with the area under the curve (AUC) of 0.816 in the training cohort.

The model displayed a high predictive ability with an AUC of 0.816 (95% CI: 0.714–0.918) in the training cohort (Fig. 4). The specificity and NPV were 0.92 and 0.96, respectively. Moreover, the AUC was 0.774 (95% CI: 0.703–0.845) in the validation cohort, which reconfirmed the model as a recommendable tool for CVD risk prediction. The website for the convenient use of the nomogram is available at https://data15651725761.shinyapps.io/DynNomapp/.

## 4. Discussion

A nomogram based on cMyBP-C, age, DBP, cigarettes per day, and family history of CVD was constructed. The model displayed a high predictive ability. Therefore, the nomogram may be a promising way for CVD risk prediction.

CVD is a widespread public health concern. Its silent development until the sudden attack poses a significant threat to the lifespan, emphasizing the crucial role of early prevention. Clinical guidelines recommend using risk prediction models to identify individuals at a high risk of CVD [22]. Over the past three decades, numerous prediction models have been developed based on various traditional risk factors. The Framingham model is the most widely used [23, 24], but a recent study demonstrated that the model overestimated the risk of CVD for the Chinese population [8]. Besides, China is experiencing an increasingly aging population, and the growing annual burden of CHD weighs mainly on the older adults aged 70–84 years [25], who are not included in the popular models such as the Framingham model [5], SCORE [6], and QRISK3 [7]. Therefore, applying these models to patients aged ≥75 years is inadequate. Furthermore, only one or no traditional risk factors are found in many patients with CVD [26]. Hence, new biomarkers are needed to improve the information gained from traditional predictors.

Multiple biomarkers that reflect myocardial injury, heart failure, and inflammation (e.g., troponin I, N-terminal pro-B-type natriuretic peptide, and C-reactive protein, respectively) have been investigated and proved to improve the predictive performance of models that include traditional risk factors of CVD [27, 28, 29, 30]. Unfortunately, a report from the Framingham Heart Study suggested that the currently selected biomarkers had only a small incremental effect on the accuracy compared with the prediction model based on traditional risk factors alone [31]. Hence, biomarker selection is crucial for improving the models based on traditional risk factors of CVD. With a lower detection limit of 0.05 ng/mL, the current cardiac troponin assay lacks the sensitivity to detect minor myocardial injury because of the low prevalence of elevated levels among healthy people. N-terminal pro-B-type natriuretic peptide is relatively well studied in primary prevention and predicts cardiovascular events; nevertheless, age contributes a powerful influence on its levels which could influence the applicable population of the prediction model. Compared with previously investigated biomarkers, cMyBP-C is produced exclusively by the myocardium and is elevated in acute MI [16] but is also detectable in all healthy individuals independent of ischemic injury [17].

The present study established a CVD risk prediction model suitable for all age groups. As expected, the nomogram incorporating age, DBP, cigarettes per day, family history of CVD, and cMyBP-C demonstrated adequate predictive ability in the training cohort (AUC = 0.816) and the validation cohort (AUC = 0.774). The novel biomarker cMyBP-C also displayed an independent association with the development of CVD. The risk prediction model consisting of cMyBP-C and traditional risk factors proved to be statistically significant; however, the AUC is not perfect, suggesting that the model can still be improved. The present study is the first to include cMyBP-C levels in a predictive model of CVD, and no comparison with previous studies was possible. Still, Tong *et al*. [18] reported an AUC of 0.629–0.638 for cMyBP-C used alone to predict future CVD, with a sensitivity of 81%–96%, specificity of 27%–48%, and NPV of 93%–97%. Next, we have proposed that combining cMyBP-C with other protein biomarkers above conventional cardiovascular risk factors could further improve risk assessment.

This study had certain limitations. First, the nomogram was derived based on a 7-year follow-up and was thus not comparable to the Framingham score. Secondly, individuals with missing data were directly excluded, which reduced the available sample size and could lead to the inaccurate predictive performance of the model [32]. Besides, the patients were all from the same hospital, inevitably leading to bias due to the relatively consistent external environment and treatment methods. A multicenter population and external validation are necessary for future derivation and validation. Another limitation was that the cMyBP-C levels were detected by ELISA. A more convenient high-sensitivity assay is essential for clinical practice. Future studies will expand the population sources and conduct external validation to optimize the prediction model in the future.

## 5. Conclusions

To the authors’ knowledge, this study is the first to propose a CVD risk prediction model including traditional risk factors and the novel biomarker cMyBP-C. The nomogram provides a quantitative approach for estimating future CVD events. Physicians and individuals can conveniently perform a CVD risk prediction through the website (https://data15651725761.shinyapps.io/DynNomapp/).

## Data Availability

The datasets are available from the corresponding author on reasonable request.

## Abbreviations

AUC, area under the receiver operating characteristic curve; Β, regression coefficient; BMI, body mass index; CHD, coronary heart disease; CI, confidence interval; Cr, creatinine; CVD, cardiovascular disease; cMyBP-C, cardiac myosin-binding protein-C; DBP, diastolic blood pressure; ELISA, enzyme-linked immunosorbent assay; FBS, fasting blood sugar; HDL-C, high-density lipoprotein cholesterol; ICD-10, International Classification of Diseases, 10th revisions; LASSO, least absolute shrinkage and selection operator; MI, myocardial infarction; NPV, negative predictive value; OR, odds ratio; SBP, systolic blood pressure; TC, total cholesterol; TG, triglyceride; UA, uric acid.

## Supplementary Material

Supplementary material associated with this article can be found, in the online version, at https://doi.org/10.31083/j.rcm2402035.
