# Supplementary material for: Development and Validation of a Nomogram to Predict the Future Risk of Cardiovascular Disease
Source: Rev Cardiovasc Med. 2023 Jan 31;24(2):35. doi: 10.31083/j.rcm2402035 (PMC11273112; doi:10.31083/j.rcm2402035)
Supplement: Supplementary file 1 [file 2153-8174-24-2-035-s1.zip › 2153-8174-24-2-035-s1.docx]

Supplementary Table 1. Basic clinical characteristics between training cohort and validation cohort.

| Variables | Training cohort(n=376) | Validation cohort(n=161) | *p* value |
| --- | --- | --- | --- |
| CVD, no/yes | 338/38 (89.9/10.1) | 145/16 (90.1/9.9) | >0.999 |
| cMyBPC, pg/mL | 26.50 [14.50, 46.50] | 26.50 [14.50, 52.00] | 0.701 |
| Age, years | 50.00 [30.00, 64.00] | 44.00 [28.00, 60.00] | 0.039 |
| Sex, female/male | 218/158 (58.0/42.0) | 83/78 (51.6/48.4) | 0.201 |
| BMI, kg/m2 | 21.20 [19.60, 22.13] | 21.00 [19.30, 21.70] | 0.064 |
| SBP, mmHg | 125.15 (16.00) | 124.45 (16.07) | 0.640 |
| DBP, mmHg | 76.00 [67.00, 83.00] | 76.00 [67.00, 81.00] | 0.990 |
| Hypertension, no/yes | 330/46 (87.8/12.2) | 138/23 (85.7/14.3) | 0.610 |
| Current smoking, no/yes | 227/149 (60.4/39.6) | 92/69 (57.1/42.9) | 0.547 |
| Cigarettes per day | 0.00 [0.00, 8.00] | 0.00 [0.00, 6.00] | 0.732 |
| FBS, mmol/L | 4.79 [4.43, 5.24] | 4.78 [4.52, 5.23] | 0.729 |
| Diabetes, no/yes | 352/24 (93.6/6.4) | 155/6 (96.3/3.7) | 0.306 |
| Cr, μmol/L | 67.21 (15.15) | 65.59 (14.64) | 0.252 |
| TC, mmol/L | 4.42 [3.78, 5.07] | 4.47 [3.75, 4.89] | 0.791 |
| TG, mmol/L | 1.72 [1.46, 1.93] | 1.67 [1.36, 1.86] | 0.048 |
| HDL-C, mmol/L | 0.97 [0.86, 1.07] | 0.97 [0.86, 1.12] | 0.552 |
| UA, μmol/L | 317.00 [257.00, 358.75] | 315.00 [263.00, 357.00] | 0.884 |
| Family history of CVD, no/yes | 292/84 (77.7/22.3) | 134/27 (83.2/16.8) | 0.179 |

CVD, cardiovascular disease; cMyBP-C, cardiac myosin-binding protein-C; BMI, body mass index; SBP, systolic blood pressure; DBP, diastolic blood pressure; FBS, fasting blood sugar; Cr, creatinine; TC, total cholesterol; TG, triglyceride; HDL-C, high-density lipoprotein cholesterol; UA, uric acid.
